# Temperature-Dependent Model of Multi-step Transcription Initiation in *Escherichia coli* Based on Live Single-Cell Measurements

**DOI:** 10.1371/journal.pcbi.1005174

**Published:** 2016-10-28

**Authors:** Samuel M. D. Oliveira, Antti Häkkinen, Jason Lloyd-Price, Huy Tran, Vinodh Kandavalli, Andre S. Ribeiro

**Affiliations:** Laboratory of Biosystem Dynamics, Department of Signal Processing, Tampere University of Technology, Tampere, Finland; National Center for Biotechnology Information (NCBI), UNITED STATES

## Abstract

Transcription kinetics is limited by its initiation steps, which differ between promoters and with intra- and extracellular conditions. Regulation of these steps allows tuning both the rate and stochasticity of RNA production. We used time-lapse, single-RNA microscopy measurements in live *Escherichia coli* to study how the rate-limiting steps in initiation of the P_lac/ara-1_ promoter change with temperature and induction scheme. For this, we compared detailed stochastic models fit to the empirical data in maximum likelihood sense using statistical methods. Using this analysis, we found that temperature affects the rate limiting steps unequally, as nonlinear changes in the closed complex formation suffice to explain the differences in transcription dynamics between conditions. Meanwhile, a similar analysis of the P_tetA_ promoter revealed that it has a different rate limiting step configuration, with temperature regulating different steps. Finally, we used the derived models to explore a possible cause for why the identified steps are preferred as the main cause for behavior modifications with temperature: we find that transcription dynamics is either insensitive or responds reciprocally to changes in the other steps. Our results suggests that different promoters employ different rate limiting step patterns that control not only their rate and variability, but also their sensitivity to environmental changes.

## Author Summary

Temperature affects the behavior of cells, such as their growth rate. However, it is not well understood how these changes result from the changes at the single molecule level. We observed the production of individual RNA molecules in live cells under a wide range of temperatures. This allowed us to determine not only how fast they are produced, but also how much variability there is in this process. Next, we fit a stochastic model to the data to identify which rate-limiting steps during RNA production are responsible for the observed differences between conditions. We found that genes differ in how their RNA production is limited by different steps and in how these are affected by the temperature, which explains why different genes respond differently to temperature fluctuations.

## Introduction

Temperature is known to affect gene expression patterns in cells. This has profound effects, as changes in transcription and translation dynamics propagate to the behavior of genetic networks, which manifests in their sensitivity to temperature changes [1–3].

The expression patterns are not solely characterized by the rates at which the genes are expressed, but also by the associated stochasticity. The latter affects the phenotypic variability of populations of genetically identical cells [4–6] and the temporal variations in the behavior of the individual cells [7]. In unicellular organisms, such variations, even at the level of single molecules [8], can determine a cell fate.

In bacteria, much of the stochasticity in gene expression stems from transcription [9]. Live-cell measurements report that different promoters and intra- and extracellular conditions result in wide differences in transcription dynamics, both in rate and stochasticity [10]. Sub-Poissonian [11], Poissonian [12], and super-Poissonian [10, 13] dynamics (featuring less, equal to, and more variability than a corresponding Poisson process, respectively) have been reported, each resulting from a different combination of mechanistic properties that shape RNA production dynamics.

The way the effects of temperature changes on transcription kinetics propagate to the cellular behavior is still poorly understood. Rates of biochemical reactions are known to be affected by temperature changes, as dictated by the laws of physics, such as the Arrhenius law; however, biochemical laws have been found to not suffice to explain changes in more complex biological processes such as bacterial growth [14].

For example, it is expected that the number of RNA polymerases, the rate at which they work, and RNA lifetimes are affected by temperature. Also, at suboptimal temperatures, *Escherichia coli* shifts to specific expression patterns by changing in transcription factor numbers [15], by regulating of the relative σ-factor concentrations [16], and by affecting DNA conformation: negative supercoiling increases at low temperatures, and relaxes at high ones [17]. Despite these findings, quantitative information of the changes and on their contribution to the changes in transcription dynamics is still lacking.

Some progress toward these goals has been made through *in vitro* measurements of the closed and open complex formation dynamics [18–20]. Another study reports that RNA polymerase, rRNA, and tRNA concentrations, and the fraction of stable RNA remained constant, while the elongation rate, ppGpp concentration, and cell growth rate increase to up to 40°C, while after 40°C the changes become complex and, e.g. the growth rate decreases [21]. So far, these studies only identified changes in mean expression rates. Further information on other dynamical properties, such as stochasticity, is required. In addition, it is unclear to what extent these measurements reflect what occurs in live cells.

Here, we study the transcription dynamics of the P_lac/ara-1_ promoter in live *E. coli* at the single RNA level, under various inducer concentrations, for a wide range of temperatures. Using statistical models, we identify the most likely changes with temperature in the rate limiting steps of transcription initiation. This expands on our previous work [22–24] in that the analysis at different temperatures allows identifying more complex underlying details of the kinetics of transcription. Also, we test if similar changes are observed in the P_tetA_ promoter. Finally, we use the inferred models to study the possible causes of the underlying changes.

## Materials and Methods

### Model of Transcription Initiation

We use the following model of transcription initiation [25], which combines the active-inactive promoter model [26] with a sequential model of transcription initiation [27, 28]:

$$\underset{\text{pre-commit}\;\text{step}}{\underset{︸}{P_{\text{off}}\overset{k_{\text{on}}}{\underset{k_{\text{off}}}{⇌}}P_{\text{on}}\overset{{\tilde{k}}_{1}R}{\to }}}I_{1}\underset{\text{post-commit}\;\text{step}}{\underset{︸}{\overset{k_{2}}{\to }I_{2}\overset{k_{3}}{\to }P_{\text{on}}+E}}$$ (1)

where *P*_off_ and *P*_on_ represent the promoter in an inactive (i.e. repressed) and active (i.e. free from repressors or bound by an activator) states, respectively, *R* represents RNA polymerases, and *I*_*k*_ are intermediate complexes of transcription initiation. Finally, the product *E* represents the elongation complex. As the RNA polymerase numbers are not observed, we let $k_{1}≐{\tilde{k}}_{1}\;R$ to represent the effective forward rate for an active gene.

In the model, the promoter switches between being active and inactive (on/off) for transcription, i.e. whether an RNA polymerase can unobstructedly reach the start site and initiate transcription [9], depending on the binding and unbinding of regulatory molecules [9, 29]. In some cases, these molecules can also affect subsequent steps [28, 30].

Note that once the promoter is in a state that allows the RNA polymerase to bind and form the closed complex, not necessarily will this result in the production of an RNA, as the closed complex can reverse to the previous state [20, 31]. As such, the system is not yet fully committed to transcription. This “full commitment” only occurs once reaching the next state (*I*_1_). At this stage, it becomes highly unlikely that any reversion occurs (e.g. in the λ PR promoter, *I*_2_ is *always* unstable compared to the complete open complex state, even at 0°C [31]).

Once this commitment occurs, it is followed by a sequence of steps responsible for the formation of a stable open complex, which includes the isomerization steps [20, 28, 31, 32]. The last step in this model represents the complex escaping the start site, clearing the promoter region. Note that the reversibility of the closed complex formation [20, 24] does not reduce the applicability of the model [24, 25]. Also, regardless of this reversibility, the model can still be separated into an *R*-dependent (pre-commit) and *R*-independent (post-commit) parts.

The ability of regulatory molecules to create an on/off promoter dynamics allows bursty RNA production when fast production events are separated by long, random off periods (due to, e.g. slow repressor unbinding) [7]. If this process dominates RNA production, the transcription intervals are highly noisy (coefficient of variation *c*_*v*_ ≥ 1). Meanwhile, if the subsequent sequential process dominates transcription, the intervals between RNA production events are more regular, resulting in less noisy transcription (*c*_*v*_ ≤ 1).

Which and how many steps most contribute to the observed transcription dynamics appears to depend on the promoter and intra- and extracellular conditions [10, 11, 13, 22, 23]. In what concerns modeling this process, for example, if the promoter’s visit to the off-state or a sequential step are fast, these can be eliminated from the model, as they do not contribute significantly to the transcription dynamics. The steps with most influence on the transcription rate are called rate limiting [32].

Here we equate the transcription intervals with those of transcription initiation, which implies that transcription elongation is neglected. This is justified by the fact that, on average, elongation is not expected to affect the transcription intervals, as each transcript is expected to be delayed by a similar time. As a result, elongation primarily adds solely extra variance in the inter-transcription intervals. However, given the timescales of elongation and transcription initiation, this additional variance can be ignored: e.g. chain elongation at 50 nt/s [33] for a 5000 nt gene is expected to add a noise term with a standard deviation of 2 s [25]. Even considering elongational pauses under GTP-starved conditions [34], the elongation noise term is therefore likely negligible at the resolution of our measurements (cf. sampling interval of 60 s). Further, we note that no differences have been found between genes with elongation regions of different length [7, 35].

As each of the steps are complex processes rather than elementary chemical reactions, it is not yet well understood how the temperature affects of each of the steps in Eq (1) in live cells. For this reason, we let each of the model parameters change as a function of the temperature according to a polynomial function, rather than according to some biochemical model. As we have relatively few samples on the temperature axis, a quadratic equation was deemed sufficient to capture either temperature-independent or a linear and nonlinear temperature-dependent relationship:

$$k_{x}{\left(T\right)}^{-1}=a_{{k_{x}}^{-1},2}\;T^{2}+a_{{k_{x}}^{-1},1}\;T+a_{{k_{x}}^{-1},0}$$ (2)

where $a_{{k_{x}}^{-1},j}$ are the order-*j* coefficients of the polynomial of the temperature-dependence of the parameter *k*_*x*_, and *T* is the temperature. While this model is not expected to provide particular insight of the mechanisms of temperature-dependence (i.e. $a_{{k_{x}}^{-1},j}$ do not encode a particular physical meaning), it allows us to identify how each parameter likely responds to temperature changes, be it independent or dependent, linear or nonlinear, or monotonic or bitonic.

The parameters of the model can be estimated from the transcription intervals in a maximum likelihood sense [25]. Confidence in the model parameters can be estimated using the delta method [36]. Further, to determine if each of the steps in the above model play a significant role in the overall dynamics, we use the Bayesian information criterion (BIC) [37] as the model selection criterion. A difference in BICs (ΔBIC) of 0 to 2, 2 to 6, or ≥ 6 indicates weak, positive, or strong evidence, respectively, against the model with the greater BIC [37]. For the purposes of BIC, a censored sample is assumed to be worth 0.5 exact samples (the exact value varies depending on how badly the sample is censored). To avoid drawing false conclusions due to this approximation, we also compute a lower bound for ΔBICs when determining if the best models fits significantly better than the alternatives (see S1 Appendix).

### Measurements and Data Acquisition

**Fig 1:**
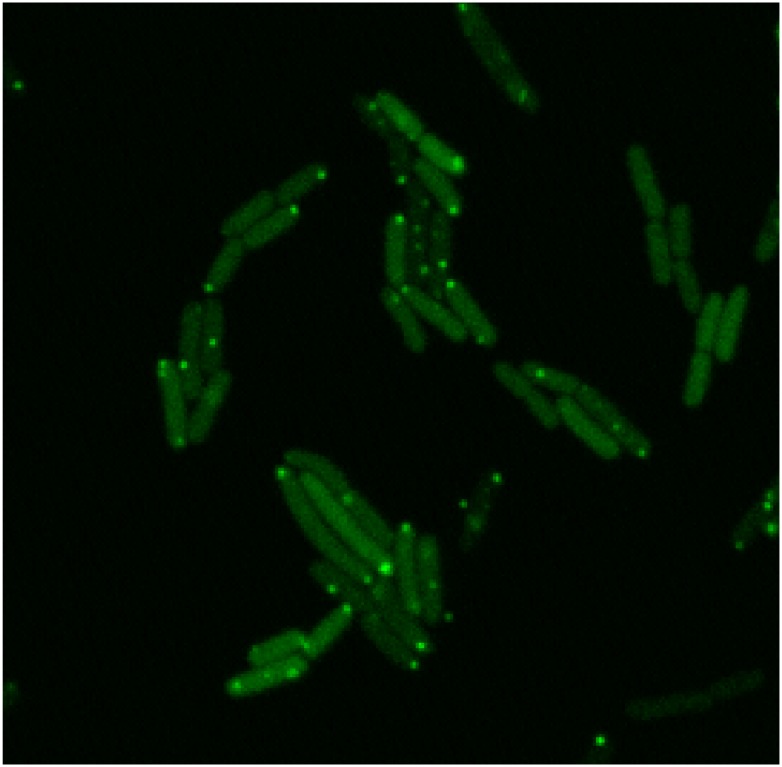
Example of a confocal microscopy image of *Escherichia coli* expressing the MS2-GPF and the target RNA. The cells are visible due the abundant MS2-GFP while the GFP-tagged target RNA appear as bright spots. The image was acquired at 35°C.

Transcription intervals in individual cells were measured in live *E. coli* using the MS2-GFP RNA-tagging system [7]. The cells feature a multi-copy reporter gene expressing MS2-GFP and a single-copy target gene, controlled by the promoter of interest, containing 96 MS2-GFP binding sites. Shortly after a target RNA is produced, the binding sites are quickly occupied by the abundant GFPs, allowing the fully tagged RNAs to be visualized using fluorescence microscopy [38]. Once formed, the RNA-96-MS2-GFP complex remains fluorescent for much longer than the cell lifetime [38]. The constructs used here were engineered previously [7, 11]. An example microscopy image is shown in Fig 1.

The activity of the target genes was also analyzed by quantitative PCR (qPCR) as a function of the media composition. To quantify intracellular RNA polymerase concentrations in each condition, we measured the amount of RpoC subunits by western blot (WB), as these are the limiting factor in the assembly of the RNA polymerase holoenzyme [39, 40]. Measuring gene activity as a function of the RNA polymerase abundance allows extrapolating the relative duration of the pre- and post-commit steps [24, 32].

More details of the constructs, measurement procedures, and data acquisition methods is given in S1 Appendix.

## Results and Discussion

### Effects of Temperature on the P_lac/ara-1_ Promoter under Various Inducer Concentrations

We studied how the distribution of durations between consecutive RNA productions in multiple, individual cells (afterwards denoted by transcription intervals) of the P_lac/ara-1_ promoter differ with temperature. This study was conducted for each possible induction scheme of this promoter, so as to assess if the temperature-dependence of the initiation kinetics is inducer scheme-dependent. Those induction schemes are: a) 1 mM of IPTG and 0.1% of arabinose (denoted by Full), b) 1 mM of IPTG only (IPTG), and c) 0.1% of arabinose only (Ara). In each condition, we recorded multiple time series of 120 minutes in length with a 1 minute sampling interval for temperatures between 24 and 41°C (see S1 Appendix for details).

**Fig 2:**
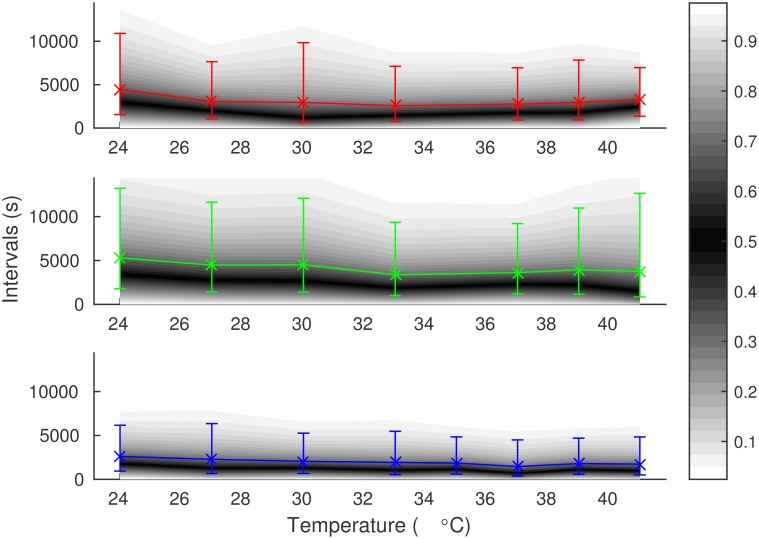
Distributions of transcription intervals in individual cells as a function of temperature for the P_lac/ara-1_ promoter. The gray gradients represent quantiles of the interval distributions, as indicated by the color bar on the right. The crosses denote means, and the error bars represent the lower and upper standard deviations of the distributions. Cases from top top bottom: IPTG (red), Ara (green), Full (blue).

We first quantified how the mean and the standard deviation (sd) of the transcription intervals change with temperature. As the cell division time varies significantly between conditions, a procedure accounting for the uneven truncation of the intervals must be used. For this, the right-censored (unobserved) transcription intervals at the end of each time series are communicated to the estimator as in [25]. First, we used gamma distribution as the model, as it can have any mean and sd independent of each other (a model must be assumed in order to estimate moments from the censored data). The results are tabulated in S1 Table. The distributions are shown in Fig 2. These data suggest that, the transcription interval duration changes by about a 2× factor along the range of temperatures tested, for each induction scheme. In addition, these changes appear to be nonlinear, and even non-monotonic in some cases. Meanwhile, the standard deviation of the transcription intervals tends to follow the mean, resulting in a coefficient of variation (*c*_*v*_) of slightly above unity with a slight increase as temperature increases. The median-to-mean ratio is approximately constant with respect to temperature at about 0.61, which indicates that the temperature affects the whole distribution and not only the long intervals.

Next, we fit the data with the model of Eq (1) independently in each induction scheme and temperature, and tested if the on/off switching and which forward steps are most responsible for the observed transcription dynamics using the Bayesian information criterion (BIC) (see Materials and Methods). The results are summarized in S2 Table. In all cases, we found evidence only for a single sequential rate limiting step. While there is weak evidence that this is true in all cases (ΔBIC lower bound (ΔBIC LB; see S1 Appendix) against any multi-step model is no less than 1.15), strong (statistically significant) evidence exists that this holds at least in certain temperature ranges (for all induction schemes, there exists a temperature with ΔBIC LB no less than 5.16). In addition, we also found evidence for the presence of on/off switching in each induction scheme, as there is a condition where the lower bound for ΔBIC is no less than 9.20, providing strong evidence that the on/off switching plays a role, in at least certain temperature ranges, regardless of the induction scheme.

As the order of the steps with rates *k*_1_, *k*_2_, *k*_3_ cannot be identified from an individual measurement by this methodology (see e.g. [25]), at this stage we cannot resolve if the rate limiting step occurs prior or after commitment to transcription. To achieve this, we combine the data from different temperatures, as models with equivalent changes in their transcription intervals can be rejected on the basis that they would require too intricate changes in their parameters between conditions (e.g. multiple parameters co-varying in a complex manner). Using this procedure, the order of the last two steps remains unknown, but the changes in each can be identified.

To combine the data, we fit the data jointly in each of the three induction schemes, such that the parameters ${k_{\text{on}}}^{-1}$, ${k_{\text{off}}}^{-1}$, ${k_{1}}^{-1}$, ${k_{2}}^{-1}$, and ${k_{3}}^{-1}$ are either a) constant, b) vary linearly, or c) vary quadratically as a function of temperature (the quadratic curve representing a nonlinear relationship). Meanwhile, no model is imposed on the changes as a function of induction scheme, as these are likely nonlinear. Again, model selection is used to determine which parameters change significantly with temperature.

We found the preferred model to be the one where *k*_on_, *k*_off_, *k*_2_, and *k*_3_ are constant and ${k_{1}}^{-1}$ is a non-linear (Full and IPTG) or linear (Ara) as a function of the temperature. The ΔBICs between the second best and the best fitting models were about 1.32, 4.92, and 1.57 (ΔBIC LBs 1.31, 4.91, and 1.30) for Ara, IPTG, and Full, respectively. Meanwhile, we found similar results in models where the step with rate *k*_3_ is removed, with ΔBICs of 1.32, 4.39, and 1.51 for Ara, IPTG, and Full, respectively, indicating that using the higher-order model does affect the identification of the temperature-dependence of the parameters.

In the best fitting models, the relationship between ${k_{1}}^{-1}$ and temperature differs under different induction schemes. However, we tested if a similar change in this parameter could explain the changes in all cases, by deriving a combined model where this parameter follows a single function, up to a scale, in the three induction schemes. We found this new model to have smaller BIC with a ΔBIC of 10.6 when compared to the best unconstrained model, (ΔBIC LB of 8.80), which provides strong evidence that the temperature affects the promoter by regulating ${k_{1}}^{-1}$ in a similar, nonlinear fashion, regardless of the induction scheme.

Finally, we tested if the changes in ${k_{1}}^{-1}$ could be explained solely by the changes in RNA polymerase numbers (cf. *R* in Eq (1)). For this, we quantified in an independent measurement the relative abundance of the RpoC subunits using western blot analysis for 24, 37, and 41°C. We found that the numbers were 0.415 and 0.562 at 24 and 41°C, respectively, relative to that of at 37°C. By plugging these numbers in our model, we found strong evidence that the parameter *k*_1_ does not follow the changes in RNA polymerase numbers (ΔBIC of 27.9, ΔBIC LB of 27.0), i.e. ${\tilde{k}}_{1}$ is not constant with temperature. Moreover, we found that if the above RNA polymerase numbers were the sole change caused by the temperature, the changes in the transcription intervals ought to be larger than what was observed *in vivo*. This suggests that there are other temperature-dependent changes in ${\tilde{k}}_{1}$, which attenuates the effects of RNA polymerases number changes.

**Fig 3:**
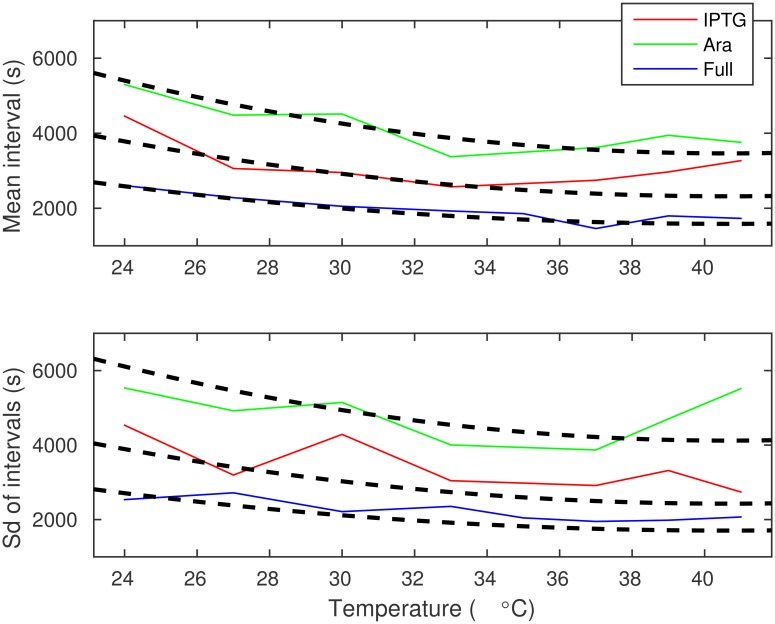
Mean and standard deviation of transcription intervals as a function of temperature for the P_lac/ara-1_ promoter. The solid curves are those shown in Fig 2. The dashed curves represent the means and standard deviations of the best fitting models.

The means and sds of the transcription intervals resulting from the best fitting model (quadratic ${k_{1}}^{-1}$ with a similar pattern in each induction scheme) are shown in Fig 3. In this model, the steps prior to transcription commitment vary between temperature conditions from 3060 to 5000 s, from 2310 to 3770 s, and from 1580 to 2590 s, while the duration of the process after transcription commitment is a constant equal to 399, 7.79, and 1.50 s for Ara, IPTG, and Full induction schemes, respectively. Interestingly, in each case, the temperature-independent post-commit step tends to be no more than 12% of the total duration of the mean interval between consecutive RNA production. Consequently, as the temperature modulates the longer lasting step in transcription, it allows for large changes in the transcription interval with temperature.

To assess whether the data fit the model as expected, we also performed Monte Carlo simulations to determine if the empirical data fits the model significantly worse than that generated from the appropriate model. For this, we generated a transcription interval from the best fitting model to correspond each sample extracted from the measurements, which was subsequently censored using the same procedure that constrains the empirical data (i.e. observation time is limited by the remaining cell lifetime, and observations occur at intervals of 60 s). This simulation was repeated for 1000 rounds, from which likelihoods were calculated. The fraction of rounds where the simulated data fit worse than the empirical data (cf. a p-value) were 0.230, 0.161, and 0.040 for Ara, IPTG, and Full, respectively, and 0.095 for all cases combined. As such, we found no evidence to reject our model (typically done e.g. when this probability is less than 0.01). The mean and sd for a single simulation is shown in S1 Fig (cf. the equivalent figure for the empirical data in Fig 3).

We compared our results on the relative durations of the pre- and post-commit steps with an independent method, namely, by extrapolating the corresponding values from a Lineweaver-Burk plot [24, 32]. For this, we used qPCR to measure the target gene activity and western blot to estimate the RNA polymerase concentrations in different media conditions (see S1 Appendix for details). We found the post-commit step to take up only about 22.5% (sd 14.6%) of the transcription duration for Full induction at 37°C. This confirms the previous result, as the values reported above, derived from the microscopy measurements, are well within the 95% confidence interval (CI; [−6.08,51.1]%) of the qPCR/WB measurements.

The parameter values for the best fitting models are listed in S3 Table and some derived features of the kinetic of the on/off switching in S4 Table. These parameter values suggest that the promoter remains most of the time unavailable for RNA polymerases to bind (small duty cycle). After this, it produces a small (≪ 1) burst, whose size is temperature-dependent. The mean interval between bursts appears to be unaffected by the removal of arabinose (cf. IPTG versus Full; as *k*_on_ is nearly constant and much smaller than *k*_off_), while removing IPTG has more complex effects. These results are consistent with observed changes in the median-to-mean ratio.

Two studies, one using fluorescent in situ hybridization (FISH) [41] and the other using a yellow fluorescent protein fusion library for *E. coli* [10] to quantify the cell-to-cell diversity in RNA numbers from several promoters, have reported Fano factors ranging from 1 to 3. Also, in the work by Golding and colleagues [7], it was estimated that the Fano factor in transcript production could be as large as 4.1. These results were taken as indicative of bursty RNA production. However, recent works have shown that much of this cell-to-cell diversity in RNA numbers can be explained by other factors [42], such as RNA degradation [43] and stochastic partitioning of RNA molecules in cell division [44]. As we base our study on the transcription intervals between consecutive RNA production events in individual cells, rather than the variability in RNA numbers, our analysis is less susceptible to such errors. In addition, only under specific conditions does a large Fano factor correspond to large burst sizes [45]. As such, we do not expect our estimate of the average burst size to be in contradiction with these studies.

### Effects of Temperature on the P_tetA_ Promoter

Even though the Lac-Ara-1 Promoter initiation kinetics responded similarly to temperature changes under all induction schemes, this response might not generalize to different promoters with different rate-limiting step configuration. To test this, we studied the effects of temperature changes on the transcription dynamics of the P_tetA_ promoter.

For this, using raw data from [11], we made use of the methodology in [25] in order to extract the single-cell transcription intervals of the P_tetA_ promoter at 24 and 37°C with 15 ng of aTc (Full induction). In each case, the time series were of about 60 minutes in length with 1 minute sampling. S5 Table tabulates the mean, sd, and *c*_*v*_ of the transcription intervals estimated from the data. The mean transcription interval changed by about a 2.5× factor.

Unlike the P_lac/ara-1_ promoter, the P_tetA_ promoter appears to have sub-Poissonian dynamics, suggesting that its RNA production kinetics is mostly dictated by a sequence of multiple rate limiting steps, rather than the activation of the promoter. To confirm this, we fit the data with the model of Eq (1) and tested for on/off switching and number of rate limiting steps. The results, shown in S6 Table, provide strong evidence for two or three rate limiting steps (ΔBIC LBs against any single step model were no less than 19.0). Meanwhile, no evidence of significant on/off switching is present (ΔBICs against best on/off model are 1.14 (LB 0.387) for 24 and 5.74 (LB 5.34) for 37°C).

Finally, we fit the two data points jointly with a 3-step model, where one of the three parameters changes as a function of the temperature (more complex changes cannot be identified with only two temperature conditions). The results suggest that the two common rate limiting steps are about 121 s each in duration, while the changing step is 1910 and 543 s for 24 and 37°C, respectively. As there is no on/off switching, we cannot identify which steps are in the pre-commit and post-commit stages. It is however possible to determine that the relative duration of the post-commit step must be either 11.3% or 94.4% for 24°C and 30.9% or 84.6% for 37°C.

To determine whether the post-commit step is the longer or shorter one, we extrapolated the relative durations of the post- and pre-commit steps from a Lineweaver-Burk plot [32] based on qPCR and WB measurements. At 37°C, we found the duration of the post-commit step to be about 77.1% (sd 6.64%) of the transcription duration with a 95% CI of [64.1,90.1]%, suggesting that, in the P_tetA_ promoter, the long, temperature-sensitive step occurs after transcription commitment.

### Modeling the Effects of Changes in the Rate Limiting Steps

Having measured the dynamics of transcription initiation of P_lac/ara-1_ and P_tet_ as a function of temperature and induction scheme, and assessed which parameter values are most responsible for the changes in the observed dynamics, we next make use of the model extracted from the data to investigate the ability of each parameter of the model in changing the dynamics of RNA production and the overall the range of behaviors possible with this model.

#### Response and bounds of mean, sd, and cv for parameter changes

We next explored the extent to which the dynamics of RNA production can change as these parameter values change. Namely, we used our model to study how changes in ${k_{\text{on}}}^{-1}$, ${k_{\text{off}}}^{-1}$, and ${k_{1}}^{-1}$ affect the mean and standard deviation (sd) of the duration of the pre-commit step. The mean, sd, and *c*_*v*_ of this step cannot be freely varied when changing *k*_on_ or *k*_off_ only. Specifically, the mean is bounded to $\left[{k_{1}}^{-1},\infty \right)$ while the variance is limited to $\left[{k_{1}}^{-2},\infty \right)$. Meanwhile, ${c_{v}}^{2}$ is bounded to [1, 1 + 2*k*_1_/*k*_off_] and to [1, 1 + *k*_1_/(2*k*_on_)] when changing *k*_on_ and *k*_off_ only, respectively. For changes in *k*_off_, *c*_*v*_ is maximized at *k*_off_ = *k*_on_, while for *k*_on_ *c*_*v*_ monotonically increases with ${k_{\text{on}}}^{-1}$. In S2 Fig, these changes in mean and sd are exemplified by varying each parameter about $\left({k_{\text{on}}}^{-1},{k_{\text{off}}}^{-1},{k_{1}}^{-1},{k_{2}}^{-1},{k_{3}}^{-1}\right)$ = (1, 1, 1, 0, 0) and computing the mean and sd.

Regardless of the dynamics of the post-commit step, the mean and sd of the total transcription interval are less tightly restricted when *k*_1_ is allowed to vary when compared to the case where only *k*_on_ or *k*_off_ varies. This results from the fact that by varying *k*_on_ or *k*_off_, the on/off switching can be disabled, but the forward step with rate *k*_1_ remains rate limiting, while for varying *k*_1_, the entire pre-commit step can be made negligible. E.g., in Eq (1) the mean is limited to ${k_{2}}^{-1}+{k_{3}}^{-1}$ versus ${k_{1}}^{-1}+{k_{2}}^{-1}+{k_{3}}^{-1}$ (cf. mean and sd < 1 for small ${k_{1}}^{-1}$ in S2 Fig).

On the mean transcription interval, the changes in *k*_on_ and *k*_off_ have an opposite effect (as the mean is $\left(1+{k_{\text{on}}}^{-1}\;/\;{k_{\text{off}}}^{-1}\right)\;{k_{1}}^{-1}+{k_{2}}^{-1}+{k_{3}}^{-1}$). Consequently, relative changes in both ${k_{\text{on}}}^{-1}$ and *k*_off_ induce an equal change in the mean (as quantified by the derivative ${k_{\text{on}}}^{-1}\;/\;{k_{\text{off}}}^{-1}\;{k_{1}}^{-1}$), while ${k_{1}}^{-1}$ always induces a greater change (derivative of $\left(1+{k_{\text{on}}}^{-1}\;/\;{k_{\text{off}}}^{-1}\right)\;{k_{1}}^{-1}$); cf. the large mean limits in S2 Fig). Whether the above changes are smaller or larger than those induced by changes in the post-commit steps (${k_{2}}^{-1}$ or ${k_{3}}^{-1}$) is determined by the relative durations of the steps. Meanwhile, the reciprocality of *k*_on_ and *k*_off_ does not extend for sd, despite the fact that at the lower bound the models become equivalent. For large ${k_{\text{on}}}^{-1}$ versus *k*_off_, the sd always changes more with relative changes in the former. These changes also differ from those induced by ${k_{1}}^{-1}$. For large values, the transcription intervals can change more with either *k*_on_ or *k*_1_, depending on the parameters, but will never change most with *k*_off_ (cf. large sd limits in S2 Fig).

**Fig 4:**
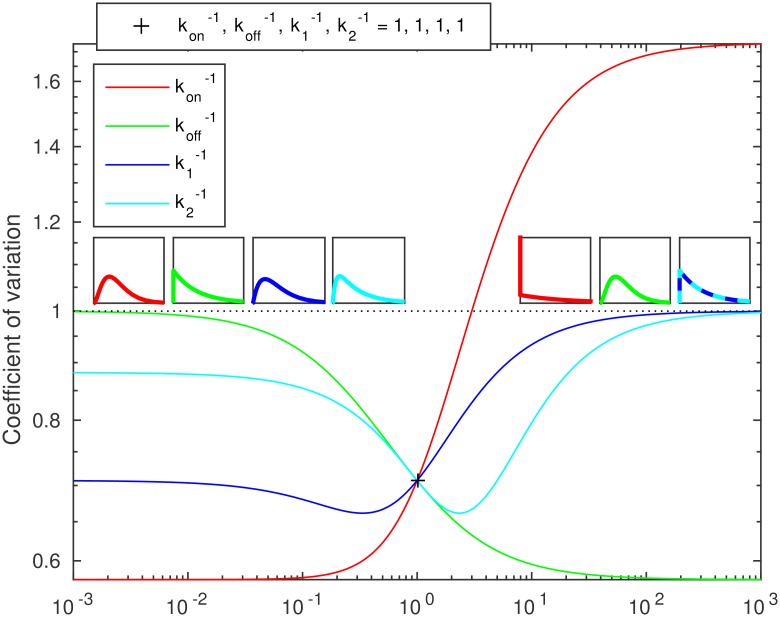
Coefficient of variation (*c*_*v*_) of transcription intervals as a function of relative changes in the parameters ${k_{\text{on}}}^{-1}$, ${k_{\text{off}}}^{-1}$, ${k_{1}}^{-1}$, and ${k_{2}}^{-1}$. The horizontal axis corresponds to the relative value in the specified parameter. The dotted line denotes a *c*_*v*_ of unity. The *c*_*v*_ at the pivot parameter is $1\;/\;\sqrt{2}$. Left (right) insets represent the asymptotic transcription interval distributions for small (large) parameter values, their color indicating the varying parameter.

While the mean and sd of the transcription intervals vary monotonically with respect to the changes in the parameters, the noise can change in an intricate manner. This is demonstrated in Fig 4. Specifically, the *c*_*v*_ can be minimized/maximized for specific values of the means and sds of both the pre-commit and post-commit steps.

In addition to the changes in *c*_*v*_ of the transcription intervals, we also studied the possible shapes of the distributions (cf. the insets of Fig 4) that can be attained by varying each parameter. The shape information is important as, for example, by varying *k*_on_, a *c*_*v*_ of unity can be attained, but in this regime the distribution necessarily lacks fast intervals (cf. exponential distribution) and consists of a population of regular intervals mixed with large outliers.

As exemplified in the insets of Fig 4, for varying ${k_{\text{on}}}^{-1}$, the distribution of transcription intervals ranges from a regular (in other words, hypoexponential, a sequence of exponentials with rates *k*_1_, *k*_2_, *k*_3_) with a low *c*_*v*_ (*c*_*v*_ < 1) to a mixed population with fast intervals combined with large outliers (highly noisy, *c*_*v*_ > 1). Meanwhile, by varying *k*_off_, the distribution ranges from regular to an exponential (*c*_*v*_ = 1), as the effects of on/off switching become time-averaged. When varying ${k_{1}}^{-1}$, the distribution ranges from that of the post-commit steps alone to an exponential with time-averaged on/off switching. However, in the latter two cases, it is possible that a highly noisy distribution can be attained for some intermediate parameter values. Alternatively, a noise minimum might exist somewhere in the intermediate region in each of the three cases. When varying ${k_{2}}^{-1}$ (or any other post-commit step), the distribution varies from a process lacking that particular step to a single exponential step with that particular rate, and a noise minimum always exists for some intermediate value where the means and sds of the steps are of specific values.

The lower bounds for the *c*_*v*_ of the transcription intervals are limited by the relative durations of the pre- and post-commit stages and number of steps in the post-commit stage. This is exemplified in S3 Fig. Generally, for a post-commit stage consisting of *n* steps and having a relative duration of *r* (relative to the mean interval between consecutive transcription events), the lower bound is (*r* − 1)^2^ + *r*^2^/*n* (cf. green curves in S3 Fig). Consequently, the lower bound for *c*_*v*_ for any value of *r* is 1/(*n* + 1) at r* = *n*/(*n* + 1), which occurs when the pre-commit step is exponential-like, and this step and all the *n* steps of the post-commit stage are of equal duration. As a result, the change in the *c*_*v*_ with changes in the rate limiting step durations is controlled by the number and relative durations of these steps during both post- and pre-commit stages.

### Exploring the Model Using the Empirical Data of P_lac/ara-1_ and P_tetA_ Promoters

Finally, we explored, in terms of the model, why the analysis identified some parameters as the most likely candidates for being responsible for the observed changes in RNA production dynamics with temperature and induction scheme, while other parameters were identified as not fit to explain these changes. In other words, we investigate the limitations of each parameter in changing the dynamics of RNA production. In this regard, note that the fitting procedure does not equate the transcription interval mean, standard deviation, nor the coefficient of variation of the model and data in particular, but maximizes the likelihood that the selected model generates the data.

**Fig 5:**
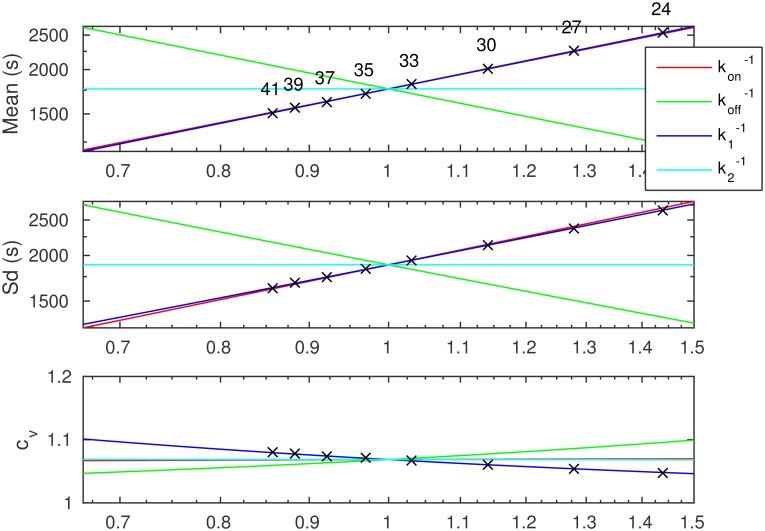
Mean, standard deviation (sd), and coefficient of variation (*c*_*v*_) of the transcription intervals when varying each parameter independently around the parameter values found for the P_lac/ara-1_ promoter, full induction. The numeric labels for the crosses indicate temperature (°C). The data points are derived from the best fitting model. The curves for ${k_{1}}^{-1}$ correspond to those shown in S4 Fig. The curve for ${k_{\text{on}}}^{-1}$ is close to that of ${k_{1}}^{-1}$, ${k_{1}}^{-1}$, and ${k_{2}}^{-1}$ for mean, sd, and *c*_*v*_, respectively. The curve for ${k_{3}}^{-1}$ is similar to that of ${k_{2}}^{-1}$ in all cases and is thus omitted.

S4 Fig shows the mean, sd, and *c*_*v*_ of the transcription intervals for the P_lac/ara-1_ as a function of temperature for the Full induction case, while Fig 5 shows the same variables in the relative parameter space about the parameters of the best-fitting model. The additional curves in Fig 5 detail the behavior of the mean, sd, and *c*_*v*_ if other parameters were changing instead. For the P_lac/ara-1_ promoter, the mean, sd, and *c*_*v*_ would not change significantly with changes in *k*_2_ and *k*_3_. This holds as *k*_2_ and *k*_3_ are relatively small (not rate limiting) and relatively large changes in them barely affect the transcription intervals. Meanwhile, while varying *k*_on_ could result in the observed changes in the mean and sd, it could not explain the observed changes in the *c*_*v*_ of the data. This is due to the fact that the model features a large ${k_{\text{on}}}^{-1}$, implying that the noise can only be effectively tuned by varying the burst size ${k_{1}}^{-1}\;/\;{k_{\text{off}}}^{-1}$. Consequently, the changes observed in the data must be explained by variations in either ${k_{\text{off}}}^{-1}$ or ${k_{1}}^{-1}$. We verified that these conclusions hold for the other model candidates as well (varying ${k_{1}}^{-1}$, ${k_{\text{off}}}^{-1}$, ${k_{2}}^{-1}$, and ${k_{3}}^{-1}$), as they could shift the model into different regions of the parameter space. In addition, we found the curves for IPTG to be similar, while for Ara, varying *k*_2_ can modulate mean and sd, but cannot explain the changes in *c*_*v*_.

Meanwhile, the corresponding curves displaying the changes in mean, sd, and *c*_*v*_ of the transcription intervals for the P_tetA_ promoter, are shown in S5 and S6 Figs. Here, both the mean and sd do not change significantly with changes in *k*_1_ and *k*_3_, the smaller two of the rate limiting steps. Again, this is due to the fact that these steps are relatively smaller. Interestingly, *k*_1_ and *k*_3_ could be used to manipulate *c*_*v*_ appropriately, which is in contrast with the behavior observed in the P_lac/ara-1_ promoter, and enabled by the more even distribution of the transcription interval durations to the pre- and post-commit stages.

Another reason why changing the identified parameters could be preferred over changing the other feasible parameters is that the two have opposite effects as a function of increasing temperature. Such results are found both in P_lac/ara-1_, where *k*_off_ and *k*_1_ have opposite effects on both mean and sd of the transcription intervals, and in P_tetA_, where *k*_1_ and *k*_2_ (or *k*_3_) have opposite effects on the *c*_*v*_. Alternatively, there could be some physical constraints e.g. on the parameters ranges which are not considered in our models.

### Conclusion

We quantified how temperature affects the dynamics (rate, stochasticity, underlying steps, etc.) of the transcription initiation kinetics of the P_lac/ara-1_ promoter in live individual *E. coli* cells. This was performed in three differing induction schemes of IPTG and arabinose, for a wide range of temperatures above and below the optimal growth temperature.

For this, we used statistical methods to compare detailed stochastic models fit to the empirical data in maximum likelihood sense. The selected models inform on the most likely way the changes in RNA production kinetics with temperature and induction scheme emerge from the changes in the rate limiting steps underlying transcription initiation. To overcome the limitations of the presently available methods of observing transcription dynamics in live cells, we performed a differential analysis of several measurements under a diverse set of conditions.

We found that not all steps in transcription initiation are affected equally by temperature changes: varying only some of them suffices to explain the changes found in the measured transcription intervals, regardless of the induction scheme. Specifically, nonlinear changes in the closed complex formation alone suffice to explain the observed changes in transcription dynamics of the P_lac/ara-1_ promoter. By correlating these changes with variations in RNA polymerase numbers, we found that these can be only partly responsible for the observed changes in transcription dynamics, which indicates that temperature affects the interaction between the transcription start site and an RNA polymerase.

Next, we used similar methods to analyze P_tetA_ promoter under two different temperatures. We found that this promoter rate-limiting events occur at different stages of transcription initiation, resulting in a different, less noisy transcription kinetics shape. In agreement, we found that its response to temperature is not explained by modulating the closed complex formation step as for the P_lac/ara-1_ promoter, but instead arises from changes in the open complex formation and/or promoter escape dynamics. Overall, this suggests that the patterns of rate-limiting step kinetics of *E. coli*’s promoters not only cause the genes to differ in RNA production rate and noise, but also in how responsive is their RNA production dynamics to temperature changes.

Finally, we used stochastic models to explore the possibilities of tuning transcription dynamics by varying each of the rate limiting steps. An advantage of modulating the identified steps over the other rate limiting steps was identified: it allows more flexibility in tuning both the mean and variance of the transcription intervals. In addition, in the region of the parameter space suggested by our data, the transcription dynamics is most sensitive to these particular changes, while other means of tuning the dynamics result in opposite changes in the response. This suggest that varying these particular steps might offer the promoters greater adaptability to temperature changes than if the transcription dynamics were tuned by other means.

Our study exemplifies how differential analysis of transcription intervals with statistical methods can inform on the underlying steps of transcription initiation which, at the moment, cannot be directly measured in live cells. We expect these techniques to be applicable, with small modifications only, to study similar processes such as translation or the behavior of genetic circuits.

## Supporting Information

S1 Appendix Extended Methods and Materials. (PDF)

S1 Table Mean, standard deviation (sd), and coefficient of variation (*c*_*v*_) of the transcription intervals for the P_lac/ara-1_ promoter. The table shows the condition, number of exact intervals (samples), number of right-censored intervals (R-samples), and the estimated mean, sd, and *c*_*v*_ of the intervals (considering exact and right-censored ones).
(CSV)

S2 Table Presence or absence of on/off switching and number of identified steps in P_lac/ara-1_ promoter. The table shows the condition, the existence or absence (yes/no) of on/off switching, the durations of significant steps (Steps 1 to 3), the second-best fitting model (Alt model), and the difference in BICs (ΔBIC) of the second-best and the best fitting models and its lower bound.
(CSV)

S3 TableEstimated model parameters for P_lac/ara-1_ promoter.The table shows the induction condition and the estimated parameter values. Here, ${k_{1}}^{-1}=A\;T^{2}+B\;T+C$, where *T* is the temperature in degrees Celsius.(CSV)

S4 Table Statistics of the on/off switching for P_lac/ara-1_ promoter. The table shows the condition, the fraction of time the gene is on (Duty cycle) and the average burst size and interval.
(CSV)

S5 Table Mean, standard deviation (sd), and coefficient of variation (*c*_*v*_) of the transcription intervals for the P_tetA_ promoter. The table shows the temperature condition, number of exact intervals (samples), number of right-censored intervals (R-samples), and the estimated mean, sd, and *c*_*v*_ of the intervals.
(CSV)

S6 Table Presence or absence of on/off switching and number of identified steps in the P_tetA_ promoter. The table shows the temperature condition, the presence or absence of on/off switching (On/off), the durations of the significant steps (Steps 1 to 3), the second-best fitting model (Alt model), the difference in BICs (ΔBIC) of the second-best and the best fitting model, and its lower bound (LB).
(CSV)

S7 Table List of promoters and measurement conditions used in the manuscript. The table shows an arbitrary condition ID, the promoter, concentrations of the inducers (IPTG (mM), Ara (L-arabinose; %), and aTc (anhydrotetracycline; ng/mL)), and the temperature (°C).
(CSV)

S8 Table List of all intervals between the production of consecutive transcripts used in the manuscript. The table shows an arbitrary interval ID, condition ID (a foreign key to), the lower (Interval LB) and upper bounds (Interval UB), and the ID of the interval that precedes in time (Previous Interval ID) for each interval observed in the cells under both promoters in each condition. The order of the rows has no particular meaning.
(CSV)

S1 Fig Mean and standard deviation of transcription intervals generated using a Monte Carlo simulation as a function of temperature for the P_lac/ara-1_ promoter. The dashed curves represent the means and standard deviations of the best fitting models.
(EPS)

S2 FigMean and standard deviation (sd) of transcription intervals as a function of the relative change in ${k_{\text{on}}}^{-1}$, ${k_{\text{off}}}^{-1}$, and ${k_{1}}^{-1}$.The horizontal axis corresponds to the relative value in the specified parameter. The dashed curves are the asymptotes discussed in the text. The pivot parameters result in a mean of 2 and sd of $\sqrt{6}$.(EPS)

S3 Fig Lower bound for the coefficient of variation of transcription intervals as a function of the relative durations between pre- and post-commit stages. Blue points represent instances of random models. Green curves are the *c*_*v*_ lower bounds for different number of steps *n* in the post-commit stage: from top to bottom: 1, 2 (solid curve; the model in Eq (1)), 3, 4, and 5. The dotted black line is the absolute lower bound attained with a constant-duration post-commit stage (*n* = ∞).
(EPS)

S4 Fig Mean, standard deviation (sd), and coefficient of variation (*c*_*v*_) of the transcription intervals for the P_lac/ara-1_ promoter under full induction, and the corresponding best fitting model. The data points correspond to those shown in S1 Table. Error bars denote one sd of the estimator uncertainty.
(EPS)

S5 Fig Mean, standard deviation (sd), and coefficient of variation (*c*_*v*_) of the transcription intervals for the P_tetA_ promoter under full induction, and the corresponding best fitting model. The data points correspond to those shown in S5 Table. Error bars denote one sd of the estimator uncertainty.
(EPS)

S6 Fig Mean, standard deviation (sd), and coefficient of variation (*c*_*v*_) of the transcription intervals when varying each parameter independently around the parameter values found for the P_tetA_ promoter, full induction. The numeric labels for the crosses indicate temperature (°C). The data points are derived from the best fitting model.
(EPS)
