# Supplementary material for: Temperature-Dependent Model of Multi-step Transcription Initiation in Escherichia coli Based on Live Single-Cell Measurements
Source: PLoS Comput Biol. 2016 Oct 28;12(10):e1005174. doi: 10.1371/journal.pcbi.1005174 (PMC5085040; doi:10.1371/journal.pcbi.1005174)
Supplement: S1 Appendix — (PDF) [file pcbi.1005174.s001.pdf]

Temperature-Dependent Model of Multi-Step Transcription Initiation in *Escherichia coli* Based on Live Single-Cell Measurements

Samuel M. D. Oliveira◑, Antti Häkkinen◑, Jason Lloyd-Price, Huy Tran, Vinodh Kandavalli, and Andre S. Ribeiro

◑These authors contributed equally to this work.

S1 Appendix: Extended Materials and Methods

Cells, Plasmids, Media, and Chemicals

The strain *E. coli* DH5α-PRO (identical to DH5α-Z1) [1], generously provided by I. Golding (Baylor College of Medicine, Houston, TX), was used to express the target and reporter plasmids. The strain genotype is deoR, endA1, gyrA96, hsdR17(rK$^-$ mK$^+$), recA1, relA1, supE44, thi-1, Δ(lacZYA-argF)U169, Φ80δlacZΔM15, F$^-$, λ$^-$, P_{N25}/tetR, P_{lacIq}/lacI, SpR. Importantly, this strain contains two constitutively overexpressed genes, *lacI* and *tetR*, under the control of P_{lacIq} and P_{N25} promoters, respectively, ensuring stable tight transcription regulation [1].

Two bacterial systems were used. The first is the mentioned strain containing: i) a medium-copy vector PROTET-K133 with the reporter gene $P_{LtetO\text{-}1}$-MS2d-GFP, which produces the dimeric fusion protein MS2d-GFP; and ii) a single-copy F-plasmid pIG-BAC, carrying the target gene $P_{lac/ara\text{-}1}$-mRFP1-MS2-96bs with a coding region for a monomeric red fluorescent protein (mRFP) followed by an array of 96 MS2 binding sites [2]. The second is a modified version of the original strain with the following differences: i) the low-copy vector pZS12 carries the reporter gene $P_{LlacO\text{-}1}$-MS2-GFP; and ii) the single-copy F-plasmid vector pIG-BAC carries the target gene P_{tetA}-mRFP1-MS2-96bs [3]. The activity of the promoters $P_{LtetO\text{-}1}$ and P_{tetA} is regulated by the repressor tetracycline (TetR) and the inducer anhydrotetracycline (aTc). Meanwhile, the activity of the promoter $P_{LlacO\text{-}1}$ is regulated by the LacI repressor and the inducer IPTG, and the activity of the promoter $P_{lac/ara\text{-}1}$ is regulated by both LacI and AraC repressors and the inducers IPTG and L-arabinose.

All strains were grown in Lysogeny Broth (LB) medium, supplemented with the appropriate antibiotics (35 µg/mL kanamycin and 34 µg/mL chloramphenicol for the first strain, and 100 µg/mL ampicillin and 34 µg/mL chloramphenicol

for the second strain). Antibiotics were from Sigma-Aldrich (USA). The composition of LB was: 10 g/L of tryptone (Sigma Aldrich, USA), 5 g/L of yeast extract (LabM, UK) and 10 g/L of NaCl (LabM, UK).

Finally, in order to obtain a set of medium conditions where differences between intracellular RNAP concentrations are maximized while differences in growth rates are minimized, we followed the procedure established in [4]. Namely, we carried out measurements in modified LB medium that have lower tryptone and yeast extract concentrations (by 0.25 or 0.5 fold), which reduces intracellular RNAP concentrations accordingly [4].

Induction of Target and Reporter Genes

Cell cultures were diluted in LB from overnight cultures to OD_{600} of 0.05, and kept at 37 °C at 250 RPM in a shaker until reaching mid-logarithmic phase with an OD_{600} of 0.3. After that, cells containing the promoters $P_{\text{lac/ara-1}}$ and $P_{\text{LtetO-1}}$ were induced with 0.1% L-arabinose and 1 mM IPTG for target activation, and 100 ng/mL aTc for reporter activation. Cells containing the target promoter P_{tetA} and the reporter promoter $P_{\text{LlacO-1}}$ were induced with 15 ng/mL aTc for target activation and 1 mM IPTG for reporter activation. In both cases, for the cells to produce sufficient MS2-GFP for the detection of target RNAs, the reporter and target genes were induced 50 minutes prior to the measurements, while keeping cells shaking at 250 RPM in the incubator at the appropriate temperature (24, 27, 30, 33, 35, 37, 39, or 41 °C). In the case of $P_{\text{lac/ara-1}}$, the induction was complemented by adding 1 mM IPTG 10 minutes prior to microscopy. In the end, cells were collected by centrifugation at $8000 \times g$ for 1 minute, and diluted in fresh LB medium. For this, 5 µL of cells were added to an agarose pad (Sigma Life Science, USA), and placed into a temperature chamber (Bioptechs, FCS2) at the appropriate temperature for image acquisition.

Microscopy

The imaging was performed using a Nikon Eclipse (Ti-E, Nikon) inverted microscope, equipped with a 100× Apo TIRF (1.49 NA, oil) objective and a C2+ (Nikon) confocal laser-scanning system. Images were captured with the aid of a motorized stage. To visualize fluorescent-tagged RNA spots, we used a 488 nm laser (Melles-Griot) and an emission filter (HQ514/30, Nikon). Time-lapse fluorescence images were taken once per minute for 120 or 60 minutes. The software used for image acquisition was NIS-Elements (Nikon), and the images were analyzed using a custom software, described below.

Image Analysis

The fluorescence microscopy images were processed as follows. First, consecutive images in the time series were aligned such that the cross-correlation of

fluorescence intensities is maximized. Next, a region occupied by each cell during the time series is manually annotated. After this, the locations, dimensions, and orientation of each cell in each frame are found by principal component analysis and the assumption that the fluorescence inside the cells is uniform [5]. Cell lineages were constructed using CellAging, which associates segments in consecutive frames based on their overlapping areas [6].

Next, the intensity of each cell is fit with a surface, which is a quadratic polynomial of the distance from the cell border, in least-deviations sense [7]. This surface is taken to represent the cellular background intensity resulting from the abundant, unbound MS2-GFPs, and is subtracted to obtain the foreground intensity. The foreground intensity is fit with a set of Gaussian surfaces, in least-deviations sense, with decreasing heights until the heights are in the 99% confidence interval of the background noise (estimated assuming a normal distribution and using median absolute deviation) [7]. The Gaussians are taken to represent fluorescent RNA spots, the volume under each representing the total spot intensity.

Since the lifetime of a MS2-GFP-tagged RNA is much longer than the cell division time [8, 3], the cellular foreground intensity is expected to be an increasing curve, with a jump corresponding to an appearance of a new tagged RNA. The jump positions are estimated using a specialized curve fitting algorithm [9]. Any observed interval between two consecutive RNA productions is recorded for further analysis as-is, while the interval occurring after the last observed production is rendered right censored [10]. These right censored data improve the accuracy and avoid underestimating the transcription duration [10], as the exactly observed intervals tend to lack more longer intervals than shorter ones.

Modeling Transcription Initiation and Transcription Intervals

We assume the model of transcription initiation specified in Eq (1) of the main manuscript. This model is a submodel of the more general model proposed in [10], which in turn combines the models of [11] and [12]. The reactions are:

$$P_{\text{off}} \underset{k_{\text{off}}}{\overset{k_{\text{on}}}{\rightleftharpoons}} P_{\text{on}} \xrightarrow{k_1} I_1 \xrightarrow{k_2} I_2 \xrightarrow{k_3} P_{\text{on}} + E \tag{1}$$

where P_{off}, P_{on}, I_1, and I_2 represent the different states of the promoter, and E represents a produced elongation complex (can be taken to approximate produced RNAs). The mechanistic details are further discussed in the main manuscript. We expect the dynamics of the above reactions to follow the stochastic chemical kinetics [13, 14].

While the phenotypic distribution of a cell population with respect to a particular gene is characterized by the RNA and protein numbers, the contribution of transcription is best characterized by the distribution of produced transcripts in a given period of time, as the former is affected by the latter along with other processes such as degradation and dilution (due to cell division) of the transcripts.

Meanwhile, the distribution of the number of produced transcripts is intimately related to the distribution of consecutive transcription intervals:

$$F_{E(t)}(k) = \mathbb{P}[\, E(t) \le k \,] = \mathbb{P}\left[\sum_{i=1}^{k+1} \tau_i > t \right] = 1 - \left({f_\tau}^{*\,k} * F_\tau \right)(t) \tag{2}$$

where $F_E(t)$ is the cumulative density of $E(t)$, f_τ and F_τ are the probability density function and cumulative density of the intervals between the production of consecutive transcripts τ_i (assumed to be independent and identically distributed), $f * g$ is the convolution of f and g, and $f^{\,*\,k}$ is the k:th convolution power of f.

For exponential $\tau \sim \mathcal{E}(\lambda)$ with rate λ, the produced RNA numbers are Poisson distributed $E(t) \sim \mathcal{P}(\lambda\, t)$, in which case it is said that the RNAs are produced according to a Poisson process, or more concisely, that the transcription is Poissonian. Regardless of the interval distribution, the two moments of the two distributions are related in the long-term limit [15]:

$$\begin{aligned} \lim_{t\to\infty} \frac{\mathbb{E}[E(t)]}{t} &= \mathbb{E}[\tau]^{-1} \\ \lim_{t\to\infty} \frac{\mathbb{V}\mathrm{ar}[E(t)]}{t} &= \mathbb{V}\mathrm{ar}[\tau]\ \mathbb{E}[\tau]^{-3} \end{aligned} \tag{3}$$

that is, the mean number of RNA produced per unit time equals the inverse of the transcription interval mean, while the Fano factor of the produced RNA numbers equals the squared coefficient (variance over squared mean) of variation of the transcription intervals. Conveniently, the latter equals always unity for Poissonian process. The long-term limit assumption is necessary such that short-term memory effects (which are present for a non-Poissonian transcription process) of the transcription process vanish.

Note that the long-term limit assumption is only necessary to link the moments of the transcription interval distribution to that of the RNA numbers as specified above, and not in determining the appropriate transcription process from the measurement data–the link in Eq (2) holds for any t.

The transcription intervals of the model of Eq (1) are conveniently written using the following functional equation:

$$f_\tau = \left(\sum_{k=0}^{\infty} \frac{k_1}{k_{\mathrm{off}} + k_1} \left(\frac{k_{\mathrm{off}}}{k_{\mathrm{off}} + k_1} \right)^{k} {f_{k_{\mathrm{off}}+k_1}}^{*\,k+1} \, {f_{k_{\mathrm{on}}}}^{*\,k} \right) * f_{k_2} * f_{k_3} \tag{4}$$

where $f_k(t) = k\, \exp(-k\, t)$ is the probability density function of the waiting time of a reaction with rate k. The parenthesized expression arises from the random number of visits to P_{off} prior to transitioning to I_1, and the latter two terms from the remaining reactions. The expression for f_τ can be simplified by manipulating it in the Laplace space ($X \mapsto \mathbb{E}\big[e^{tX}\big]$) [10]. The result can be

written as:

$$
\begin{aligned}
f_\tau(t) &= \sum_{j=1}^{n} \frac{k_{\text{on}} - p_j}{k_{\text{on}}} \left(\prod_{i=1}^{n} \frac{p_i}{p_i - p_j} \right) p_j \, \exp(-p_j \, t) \\
\text{where} \quad p_1, \, p_2 &= \frac{k_{\text{off}} + k_1 + k_{\text{on}}}{2} \pm \frac{\sqrt{(k_{\text{off}} + k_1 - k_{\text{on}})^2 + 4 \, k_{\text{on}} \, k_{\text{off}}}}{2} \\
p_3 &= k_2, \, p_4 = k_3
\end{aligned}
\tag{5}
$$

provided that k_{on}, p_1, $\cdots$, p_n are distinct. The singularities $p_i = p_j$ can be removed, and a more general result can be found in our previous article [10]: essentially, in such case, the density is a mixture of Erlang densities instead of the exponential ones as above.

From the above equation, several choices of parameters can result in an identical distribution of transcription intervals (and, consequently, RNA numbers). For example, k_2 and k_3 can be interchanged. This implies that while the best fitting distribution for a set of data can be found, the order of the steps k_2 and k_3, or in fact, the exact values of any of the parameters, cannot be identified without additional information. In the manuscript, we exploit the details of how the parameters change to provide such additional information to identify k_{off}, k_{on}, and k_1.

The other properties of the transcription intervals can be derived from the density, e.g. the mean and variance of the transcription intervals can be integrated from f_τ:

$$
\begin{aligned}
\mathbb{E}[\tau] &= \left(1 + \frac{k_{\text{off}}}{k_{\text{on}}}\right) k_1{}^{-1} + k_2{}^{-1} + k_3{}^{-1} \\
\mathbb{V}\text{ar}[\tau] &= \left(\left(1 + \frac{k_{\text{off}}}{k_{\text{on}}}\right)^2 + 2 \, \frac{k_1}{k_{\text{on}}} \, \frac{k_{\text{off}}}{k_{\text{on}}} \right) k_1{}^{-2} + k_2{}^{-2} + k_3{}^{-2}
\end{aligned}
\tag{6}
$$

which can alternatively be derived from the results of Peccoud and Ycart [12] using Eq (3), which links the moments of the long-term RNA distribution to those of the transcription intervals.

The duty cycle, average burst size, and average burst interval of the on/off switching loop are $k_{\text{on}} \, / \, (k_{\text{on}} + k_{\text{off}})$, $k_1 \, / \, k_{\text{off}}$, and $k_{\text{on}}{}^{-1} + k_{\text{off}}{}^{-1}$, respectively. Here, the duty cycle is the fraction of time the gene spends in the on versus off state, the burst size is the number of RNAs produced prior to turning off, and the burst interval is the duration between such bursts.

In the temperature-dependent models, each model parameter changes as a function of temperature according to a polynomial function:

$$
k_x(T)^{-1} = \sum_{j=0}^{p} a_{k_x{}^{-1}, j} \, T^j \tag{7}
$$

where $a_{k_x{}^{-1}, j}$ is the order-j coefficient for the parameter k_x and T is temperature. In practice, we consider polynomials up to the second order $p = 2$, as an

order-p polynomial (or higher) can always pass through to $p+1$ data points. We do not expect the polynomial models to provide particular insight, rather, parameters with a model of orders 0, 1, and 2, indicate that the parameter is independent of, varies linearly, or in a nonlinear fashion, respectively, with temperature.

Model Fitting and Selection

The models are fit using censored time intervals between the production of consecutive transcripts extracted from live-cell measurements (intervals are available in S8 table). The censoring is necessary, as production intervals longer than cell division cannot be observed, resulting in underestimation of the true transcription intervals, and as it improves the accuracy of the parameter estimation by properly accounting for the effects of finite sampling rate (60 s sampling interval) [10]. With censoring, rather than observing the production intervals τ_i, we observe bounds for each interval: $\tau_i \in [x_i, y_i]$.

The models are fit in a maximum likelihood sense. The maximum likelihood estimate is:

$$\begin{aligned}\hat{\boldsymbol{\theta}} &\doteq \arg\max_{\boldsymbol{\theta}} \mathbb{P}[\ \tau_1 \in [x_1, y_1], \cdots, \tau_m \in [x_m, y_m] \,|\, \boldsymbol{\theta}\ ] \\ &= \arg\max_{\boldsymbol{\theta}} \underbrace{\sum_{i=1}^{m} \log(\, F_\tau(y_1|\boldsymbol{\theta}) - F_\tau(x_1|\boldsymbol{\theta})\,)}_{\tilde{\ell}(\boldsymbol{\theta})}\end{aligned} \tag{8}$$

where $\boldsymbol{\theta}$ represents a vector of the parameters to be estimated. If multiple models are to be fit together with independent data sets, the likelihoods sum as for the different samples above. The parameter vector $\boldsymbol{\theta}$ contains the appropriate set of the polynomial coefficients $a_{k_x,j}$ that determine the model parameters for each temperature T. Here, the objective $\tilde{\ell}$ is some function that is equal up to some additive constant to the logarithm of the likelihood function ℓ.

In general, the maximum likelihood objective is not guaranteed to feature attractive properties such as convexity or unimodality, but it is smooth almost everywhere and in practice well behaved, provided that the model is somewhat correct. The optimization was performed using a general-purpose derivative-free nonlinear optimization algorithm [16]. To counter the convergence of the optimization procedure to a local maximum, we used 1,000 random restarts, with each parameter being generated from an unit-interval uniform distribution. The parameters were scaled to have a mean equal to that of the data, assuming that the data were exponential.

The distribution of the estimated parameters or any model feature derived from them can be estimated using the delta method. It can be shown that a mapping applied to the maximum likelihood estimate converges in distribution to that applied to the true parameter such that [17]:

$$\sqrt{m}\left(\mathbf{g}(\hat{\boldsymbol{\theta}}) - \mathbf{g}(\boldsymbol{\theta})\right) \xrightarrow{\mathrm{d}} \mathcal{N}\Big(\mathbf{0}, \mathbf{g}_{\boldsymbol{\theta}}(\boldsymbol{\theta})\, \mathcal{I}(\boldsymbol{\theta})^{-1}\, \mathbf{g}_{\boldsymbol{\theta}}(\boldsymbol{\theta})^T\Big) \tag{9}$$

for any $\mathbf{g}(\boldsymbol{\theta})$ that is continuous almost everywhere. Here, $\mathbf{g}_{\boldsymbol{\theta}}$ is the Jacobian of $\mathbf{g}$, $\mathcal{I}(\boldsymbol{\theta})$ is the Fisher information matrix, and $\cdot \xrightarrow{\mathrm{d}} \mathcal{N}(\mathbf{b}, \mathbf{c})$ represents a convergence in distribution to a normal distribution with a mean of $\mathbf{b}$ and covariance $\mathbf{c}$. For practical purposes, the Jacobian $\mathbf{g}_{\boldsymbol{\theta}}(\boldsymbol{\theta})$ can be approximated with that of at the parameter estimate $\mathbf{g}_{\boldsymbol{\theta}}(\hat{\boldsymbol{\theta}})$, and the Fisher information at the true parameter $\boldsymbol{\theta}$ can be approximated with the observed information $-\ell_{\boldsymbol{\theta}\boldsymbol{\theta}}(\hat{\boldsymbol{\theta}})$ at the parameter estimate, where $\ell(\boldsymbol{\theta})$ is the logarithm of the likelihood function and $\ell_{\boldsymbol{\theta}\boldsymbol{\theta}}$ its Hessian. In this work, $\mathbf{g}_{\boldsymbol{\theta}}(\hat{\boldsymbol{\theta}})$ is computed using automatic differentiation and $\ell_{\boldsymbol{\theta}\boldsymbol{\theta}}(\hat{\boldsymbol{\theta}}) = \tilde{\ell}_{\boldsymbol{\theta}\boldsymbol{\theta}}(\hat{\boldsymbol{\theta}})$ is computed numerically.

As the models with more parameters fit never worse than those with less, a scheme to penalize the excess degrees of freedom in the models is required. For this, we use the Bayesian information criterion (BIC) [18]. The BIC is computed according to [18]:

$$\mathrm{BIC} \doteq -2\,\ell(\hat{\boldsymbol{\theta}}) + k\,\log(n) \tag{10}$$

where $\ell(\hat{\boldsymbol{\theta}})$ is the log-likelihood at the maximum likelihood estimate, k is the number of parameters, and n is the number of samples. In general, $\ell(\hat{\boldsymbol{\theta}})$ and n are not known when some of the data are censored. Instead, we know $\tilde{\ell}(\hat{\boldsymbol{\theta}})$, the log-likelihood up to some additive constant, and n is known to be in some range, as each censored interval can contain information worth of 0 to 1 samples (the specific value depending on both the sample and the true model, and as such, cannot be determined). However, the additive constant vanishes when comparing two BICs, so the difference of two BICs can be estimated as:

$$\Delta\widehat{\mathrm{BIC}}_{1,2} \doteq \widehat{\mathrm{BIC}}_1 - \widehat{\mathrm{BIC}}_2 = -2\left(\tilde{\ell}_1(\hat{\boldsymbol{\theta}}_1) - \tilde{\ell}_2(\hat{\boldsymbol{\theta}}_2)\right) + (k_1 - k_2)\,\log(\hat{n}) \tag{11}$$

where $\hat{n} \doteq 1\,n_i + 0.5\,n_r$ is an estimate of the effective number of samples. As indicated above, in this work, each of the n_i interval censored samples is assumed to be worth of 1 samples, as the sampling intervals are relatively short compared to the transcription intervals, and each of the n_r right censored sample is assumed to be worth of 0.5 samples.

Finally, a conservative lower bound for $\Delta\mathrm{BIC}_{1,2}$ can be derived:

$$\Delta\mathrm{BIC\ LB}_{1,2} \doteq \min_{n \in [n_i, n_i + n_r]} -2\left(\tilde{\ell}_1(\hat{\boldsymbol{\theta}}_1) - \tilde{\ell}_2(\hat{\boldsymbol{\theta}}_2)\right) + (k_1 - k_2)\,\log(n) \tag{12}$$

which guarantees that invalid conclusions are not drawn due to the inaccuracy of the approximation, and allows a degree of inaccuracy in the former.

qPCR of Target Gene Activity

The activity of the target genes were also analyzed using quantitative PCR (qPCR). Cells containing the target plasmids were grown at various LB media [4] at 37 °C, and induced with their respective inducers (0.1% L-arabinose and 1 mM IPTG for $\mathrm{P_{lac/ara\text{-}1}}$-mRFP1-96BS, and 15 ng/mL aTc for $\mathrm{P_{tetA}}$-mRFP-96BS), as described above. Cells were collected by centrifugation at $8000 \times g$

for 5 minutes. Twice the cell culture volume of RNA protect reagent (Qiagen) were added to the reaction tube, following the addition of Tris EDTA lysozyme buffer (pH 8.0) for enzymatic lysis. The total RNA from cells was isolated by the RNeasy kit (Qiagen), according to the manufacturer instructions. The concentration of RNA was quantified by Nanovue plus spectrophotometer (GE Healthcare). To remove the residual DNA, the samples containing the total isolated RNA samples were treated with DNase. Following that, iSCRIPT reverse transcription super mix was added for cDNA synthesis. Next, the cDNA samples were mixed with the qPCR master mix, containing iQ SYBR Green supermix (Biorad), with specific primers for the target and reference genes. The reaction was carried out in triplicates with a total reaction volume of 20 µL. For quantifying the target gene, we used mRFP1 primers (forward: 5' TACGACGCCGAGGTCAAG 3' and reverse: 5' TTGTGGGAGGTGATGTCCA 3') and for the reference gene, we used the 16S RNA primers (forward: 5' CGTCAGCTCGTGTTGTGAA 3' and reverse: 5' GGACCGCTGGCAACAAAG 3'). The qPCR experiments were performed using a MiniOpticon Real time PCR system (Biorad). The following conditions were used during the reaction: 40 cycles at 95 °C for 10 s, 52 °C for 30 s, and 72 °C for 30 s for each cDNA replicate. We used no-RT controls and no-template controls to crosscheck non-specific signals and contamination. PCR efficiencies of the reactions were greater than 95%. The data from CFX Manager TM software was used to calculate the relative gene expression and its standard error [19].

Western Blot for RNA Polymerase Quantification

To quantify RNA polymerase abundance in DH5α-PRO strain at the different media, we measured the amount of RpoC subunits by western blot. Cells were grown until reaching mid-logarithmic phase, and harvested by centrifugation at $8000 \times g$ for 1 minute. After that, cell lysate was treated with the B-PER bacterial protein extraction reagent (Thermo scientific), containing protease inhibitors, and incubated at room temperature for 10 minutes. The samples were centrifuged at $15000 \times g$ for 10 minutes, after which the supernatant was collected. Next, the total protein samples were diluted to the 4× lamellae sample loading buffer, containing β-mercaptoethanol, and boiled for 5 minutes at 95 °C. The samples containing about 30 µg of total protein were resolved by 4 to 20% TGX stain free precast gels (Biorad). Proteins were separated by electrophoresis and then electro-transferred on the PVDF membrane. Membranes were blocked with 5% non-fat milk and incubated with primary RpoC antibodies of 1 : 2000 dilutions (Biolegend) overnight at 4 °C, followed by the HRP-secondary antibodies 1 : 5000 dilutions (Sigma Aldrich) for 60 minutes at room temperature. For detection of the RpoC protein, chemilumiscence reagent (Biorad) was used. Images were generated by the Chemidoc XRS system (Biorad). Band quantification was done by using the Image Lab software (version 5.2.1).

References

[1] Lutz R, Bujard H. Independent and tight regulation of transcriptional units in *Escherichia coli* via the LacR/O, the TetR/O and AraC/I1-I2 regulatory elements. Nucl Acids Res. 1997;25(6):1203–1210.

[2] Golding I, Paulsson J, Zawilski SM, Cox EC. Real-time kinetics of gene activity in individual bacteria. Cell. 2005;123(6):1025–1036.

[3] Muthukrishnan AB, Kandhavelu M, Lloyd-Price J, Kudasov F, Chowdhury S, Yli-Harja O, et al. Dynamics of transcription driven by the tetA promoter, one event at a time, in live *Escherichia coli* cells. Nucl Acids Res. 2012;40(17):8472–8483.

[4] Lloyd-Price J, Startceva S, Kandavalli V, Chandraseelan JG, Goncalves N, Oliveira SMD, et al. Dissecting the stochastic transcription initiation process in live *Escherichia coli*. DNA Res. 2016;23(3):203–214.

[5] Kandhavelu M, Hakkinen A, Yli-Harja O, Ribero AS. Single-molecule dynamics of transcription of the lar promoter. Phys Biol. 2012;9(2):026004.

[6] Hakkinen A, Muthukrishnan AB, Mora A, Fonseca JM, Ribeiro AS. CellAging: a tool to study segregation and partitioning in division in cell lineages of *Escherichia coli*. Bioinformatics. 2013;29(13):1708–1709.

[7] Hakkinen A, Kandhavelu M, Garasto S, Ribeiro AS. Estimation of fluorescence-tagged RNA numbers from spot intensities. Bioinformatics. 2014;30(8):1146–1153.

[8] Golding I, Cox EC. RNA dynamics in live *Escherichia coli* cells. Proc Natl Acad Sci USA. 2004;101(31):11310–11315.

[9] Hakkinen A, Ribeiro AS. Estimation of GFP-tagged RNA numbers from temporal fluorescence intensity data. Bioinformatics. 2015;31(1):69–75.

[10] Hakkinen A, Ribeiro AS. Characterizing rate limiting steps in transcription from RNA production times in live cells. Bioinformatics. 2016;32(9):1346–1352.

[11] McClure WR. Rate-limiting steps in RNA chain initiation. Proc Natl Acad Sci USA. 1980;77(10):5634–5638.

[12] Peccoud J, Ycart B. Markovian Modelling of Gene Product Synthesis. Theor Pop Biol. 1995;48:222–234.

[13] McQuarrie DA. Stochastic approach to chemical kinetics. J Appl Prob. 1967;4(3):413–478.

[14] Gillespie DT. A rigorous derivation of the chemical master equation. Physica A. 1992;188(1–3):404–425.

[15] Cox DR. Renewal theory. London, UK: Methuen; 1962.

[16] Nelder JA, Mead R. A simplex method for function minimization. Comput J. 1965;7(4):308–313.

[17] Lehmann EL, Casella G. Theory of point estimation. 2nd ed. New York, NY: Springer-Verlag; 1998.

[18] Kass RE, Raftery AE. Bayes factors. J Am Stat Assoc. 1995;90(430):773–795.

[19] Livak KJ, Schmittgen TD. Analysis of relative gene expression data using real-time quantitative PCR and the $2^{-\Delta\Delta C_T}$ method. Methods. 2001;25(4):402–408.
